# Initial and Repeated Point Prevalence Surveys to Inform SARS-CoV-2 Infection Prevention in 26 Skilled Nursing Facilities — Detroit, Michigan, March–May 2020

**DOI:** 10.15585/mmwr.mm6927e1

**Published:** 2020-07-10

**Authors:** Guillermo V. Sanchez, Caitlin Biedron, Lauren R. Fink, Kelly M. Hatfield, Jordan Micah F. Polistico, Monica P. Meyer, Rebecca S. Noe, Casey E. Copen, Amanda K. Lyons, Gonzalo Gonzalez, Keith Kiama, Mark Lebednick, Bonnie K. Czander, Amen Agbonze, Aimee R. Surma, Avnish Sandhu, Valerie H. Mika, Tyler Prentiss, John Zervos, Donia A. Dalal, Amber M. Vasquez, Sujan C. Reddy, John Jernigan, Paul E. Kilgore, Marcus J. Zervos, Teena Chopra, Carla P. Bezold, Najibah K. Rehman

**Affiliations:** ^1^CDC COVID-19 Response Team; ^2^Detroit Health Department, Detroit, Michigan; ^3^Detroit Medical Center, Detroit, Michigan; ^4^Wayne State University, Detroit, Michigan; ^5^Henry Ford Health System, Detroit, Michigan.

Skilled nursing facilities (SNFs) are focal points of the coronavirus disease 2019 (COVID-19) pandemic, and asymptomatic infections with SARS-CoV-2, the virus that causes COVID-19, among SNF residents and health care personnel have been described (*1*–*3*). Repeated point prevalence surveys (serial testing of all residents and health care personnel at a health care facility irrespective of symptoms) have been used to identify asymptomatic infections and have reduced SARS-CoV-2 transmission during SNF outbreaks (*1*,*3*). During March 2020, the Detroit Health Department and area hospitals detected a sharp increase in COVID-19 diagnoses, hospitalizations, and associated deaths among SNF residents. The Detroit Health Department collaborated with local government, academic, and health care system partners and a CDC field team to rapidly expand SARS-CoV-2 testing and implement infection prevention and control (IPC) activities in all Detroit-area SNFs. During March 7–May 8, among 2,773 residents of 26 Detroit SNFs, 1,207 laboratory-confirmed cases of COVID-19 were identified during three periods: before (March 7–April 7) and after two point prevalence surveys (April 8–25 and April 30–May 8): the overall attack rate was 44%. Within 21 days of receiving their first positive test results, 446 (37%) of 1,207 COVID-19 patients were hospitalized, and 287 (24%) died. Among facilities participating in both surveys (n = 12), the percentage of positive test results declined from 35% to 18%. Repeated point prevalence surveys in SNFs identified asymptomatic COVID-19 cases, informed cohorting and IPC practices aimed at reducing transmission, and guided prioritization of health department resources for facilities experiencing high levels of SARS-CoV-2 transmission. With the increased availability of SARS-CoV-2 testing, repeated point prevalence surveys and enhanced and expanded IPC support should be standard tools for interrupting and preventing COVID-19 outbreaks in SNFs.

From mid-March through early April, rapid increases in confirmed COVID-19 cases were detected among SNF residents in Detroit. During March 7–April 7, limited SARS-CoV-2 testing capacity resulted in prioritization of symptomatic residents for testing. Expansion of the Detroit Health Department testing capacity in early April enabled testing of Detroit residents from all 26 SNFs who had not previously been tested. Any testing conducted during April 8–25 was considered part of the first point prevalence survey. After the first survey, 12 facilities were prioritized for a second survey, in which participation was determined by the proportion of positive results from the first survey and the feasibility of conducting repeat on-site testing. The second survey occurred on a single date at each facility during April 30–May 8.

A Detroit Health Department rapid-testing clinic was established on April 2, 2020, using the Abbott ID NOW molecular COVID-19 test (*4*). During the first point prevalence survey, specimens collected from residents’ anterior nares were tested using the point-of-care platform in the Detroit Health Department rapid-testing clinic. Because of limited test availability for this platform, testing of specimens for the second survey was performed by an off-site reference laboratory using nasopharyngeal specimens and the SARS-CoV-2 real-time reverse transcription–polymerase chain reaction (RT-PCR) assay. At two facilities, anterior nares specimens for the second survey were collected and sent to a different reference laboratory for real-time RT-PCR testing. All specimens were collected, transported, and tested in accordance with CDC recommendations (*5*).

On-site IPC assessments and consultation were provided to facility leaders in all 26 SNFs during the first survey. Two follow-up IPC assessments were conducted for the 12 facilities participating in the second survey and included examination of cohorting practices using a facility floorplan, supply and use of personal protective equipment, hand hygiene practices, staffing mitigation planning, and other IPC activities.

Individual-level data on positive test dates, symptom status, hospitalizations, and fatalities were collected from Detroit Health Department COVID-19 case investigations, laboratory requisition forms, cases reported to the Michigan Department of Health and Human Services, and a review of death certificates. Symptom information at the time of testing was collected by oral report from facility nurse managers or from documentation of resident symptom screening. Hospitalizations included those with admission dates 2 days before through 21 days after the collection of a specimen with a positive test result for SARS-CoV-2, and deaths included those occurring within 21 days of collection of a positive specimen. To identify ongoing transmission, facility-level percentages of newly identified cases (residents with newly diagnosed SARS-CoV-2 infection divided by total number of residents tested without previous positive test results) were compared across facilities for each of the survey periods. Data were collected as part of public health response activities and were determined by CDC not to constitute human subject research.* Persons provided consent for testing and symptom screening, consistent with the policies of the facility. Analyses were conducted using SAS software (version 9.4; SAS Institute).

During March 7–May 8, among 2,773 Detroit SNF residents, 1,207 (44%) laboratory-confirmed COVID-19 cases were identified (Table). Among residents with positive test results, the median patient age was 72 years (interquartile range [IQR] = 64–82 years), 446 (37%) were hospitalized, and 287 (24%) died (Figure), including 233 (52%) hospitalized patients. Among 1,027 COVID-19 patients with symptom data available, 566 (55%) were symptomatic at the time of their first positive test result; this was highest before the first point prevalence survey (93%), decreased to 48% in the first survey, and decreased further to 4% in the second survey. Among 566 COVID-19 patients who reported symptoms, 227 (40%) died within 21 days of testing, compared with 25 (5%) among 461 patients who reported no symptoms; 35 (19%) deaths occurred among 180 patients for whom symptom status was unknown. Before the first survey, 332 residents had positive SARS-CoV-2 test results (range = 2–32 per facility). The median interval from first documented symptom onset in a facility until the first survey was 33 days (range = 20–44 days). The average facility census during the time of the first survey (April 8–25) was 96 residents (range = 38–169). During this time, 716 residents (32%) received a positive SARS-CoV-2 test result among 2,218 who had not previously received a positive test result; facilities each identified six to 77 residents with newly diagnosed infections (range = 7%–58% of residents).

**TABLE:** Initial and follow-up point prevalence survey test results for Detroit skilled nursing facility residents before the survey period, at the first survey, and at the second survey — Detroit, April–May 2020

Facility	Total tested, no.	Total positive, no. (%)	Hospitalized,* no. (%)	Died,* no. (%)	Pre-survey		First survey			Second survey		
					(March 7–April 7)		(April 8–25)			(April 30–May 8)		
					Positive, no.	Symptomatic, %	Tested,^†^ no.	Positive, no. (%)	Symptomatic, %	Tested,^†^ no.	Positive, no. (%)	Symptomatic, %
All	2,773	**1,207 (44)**	**446 (37)**	**287 (24)**	**332**	**93**	**2,218**	**716 (32)**	**48**	**637**	**115 (18)**	**4**
A	185	91 (49)	35 (38)	20 (22)	31	97	122	39 (32)	38	80	19 (24)	5
B	166	87 (52)	37 (43)	23 (26)	32	97	108	35 (32)	60	75	19 (25)	11
C	137	61 (45)	15 (25)	6 (10)	2	100	115	46 (40)	18	68	12 (18)	0
D	118	24 (20)	18 (75)	11 (46)	16	100	87	6 (7)	83	64	2 (3)	50
E	137	75 (55)	40 (53)	24 (32)	27	100	102	29 (28)	61	59	18 (31)	0
F	97	51 (53)	11 (22)	10 (20)	14	100	76	23 (30)	22	54	13 (24)	8
G	98	31 (32)	5 (16)	3 (10)	3	100	76	20 (26)	100	51	8 (16)	0
H	175	105 (60)	31 (30)	23 (22)	22	95	139	77 (55)	47	48	5 (10)	0
I	100	52 (52)	19 (37)	14 (27)	16	88	66	29 (44)	36	48	5 (10)	0
J	121	68 (56)	18 (26)	14 (21)	26	92	80	35 (44)	41	42	7 (17)	0
K	61	26 (43)	10 (38)	6 (23)	3	100	55	19 (35)	100	29	3 (10)	0
L	51	26 (51)	8 (31)	2 (8)	7	71	37	15 (41)	20	19	4 (21)	0
M	161	34 (21)	20 (59)	14 (41)	10	90	151	24 (16)	47	—^§^	—	—
N	122	36 (30)	9 (25)	9 (25)	7	100	112	27 (24)	100	—	—	—
O	122	44 (36)	24 (55)	13 (30)	18	83	97	24 (25)	50	—	—	—
P	109	40 (37)	15 (38)	7 (18)	12	92	88	21 (24)	37	—	—	—
Q	106	67 (63)	16 (24)	12 (18)	15	67	85	38 (45)	73	—	—	—
R	100	29 (29)	14 (48)	12 (41)	13	92	86	16 (19)	44	—	—	—
S	87	32 (37)	16 (50)	11 (34)	16	93	66	15 (23)	36	—	—	—
T	85	14 (16)	8 (57)	3 (21)	8	Unknown	77	6 (8)	Unknown	—	—	—
U	83	55 (66)	18 (33)	12 (22)	14	86	66	38 (58)	89	—	—	—
V	79	48 (61)	24 (50)	15 (31)	5	100	73	41 (56)	72	—	—	—
W	80	36 (45)	7 (19)	6 (17)	2	50	77	34 (44)	26	—	—	—
X	75	26 (35)	13 (50)	4 (15)	4	100	68	19 (28)	42	—	—	—
Y	64	34 (53)	10 (29)	7 (21)	3	100	61	31 (51)	13	—	—	—
Z	54	15 (28)	5 (31)	6 (38)	6	100	48	9 (19)	50	—	—	—

* Hospitalizations with admission dates documented as 2 days before, through 21 days after, the specimen collection date for a positive SARS-CoV-2 test result were counted; deaths within 21 days of positive specimen collection date were counted. Missing dates were considered to be within 21 days of specimen collection.

^†^ Total tested refers to residents tested at any time through May 8, 2020. Tested refers to residents tested in each period who were not previously known to have SARS-CoV-2 infection.

^§^ Dashes indicate that facilities did not participate in the follow-up survey.

**FIGURE:**
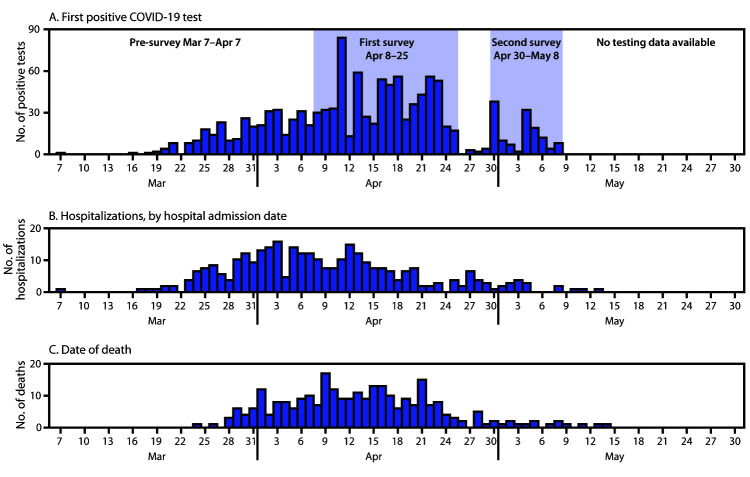
Skilled nursing facility residents with confirmed COVID-19 diagnosed by May 8, 2020, (A) by date of first positive SARS-CoV-2 test result (n = 1,190)*; (B) date of hospital admission (n = 331)^†^,^§^; and (C) date of death (n = 282)^§^,^¶^ — 26 facilities,** Detroit, March 7–May 29, 2020 **Abbreviation:** COVID-19 = coronavirus disease 2019. * Seventeen dates of first positive test results are not known. † Five residents had multiple admissions; 120 had unknown hospitalization dates. ^§^ Hospitalization and mortality data were current as of May 29, 2020. Hospitalizations with admission dates documented as 2 days before, through 21 days after the specimen collection date for a positive SARS-CoV-2 test were counted; deaths within 21 days of positive specimen collection date were counted. ¶ Five dates of death are not known. ** Data from all 26 facilities are displayed; only 12 facilities were tested during the second survey. COVID-19 testing data are not shown after May 8.

Among the 12 facilities participating in the second point prevalence survey during April 30–May 8, eight had implemented cohorting of residents with positive test results in a dedicated COVID-19 unit before the first survey; the remaining four facilities initiated cohorting shortly after receiving results from the first survey. Four of 12 facilities that took part in the second survey did not dedicate health care personnel to exclusively care for residents within the COVID-19 unit, primarily because of staffing shortages.

The average census of facilities participating in the second survey was 80 residents (range = 36–147), and 373 of 1,063 (35%) residents had received positive test results during the first survey. Among 637 residents tested during the second survey who were not previously known to have COVID-19, 18% (115) had positive SARS-CoV-2 test results; including 17% (85 of 491) of residents whose test results during the first survey had been negative. The median interval between the first and second surveys was 15 days (IQR = 14–17 days). Facilities identified two to 19 new cases during the second point prevalence survey (range = 3%–31% of residents tested).

## Discussion

Facility-wide testing conducted among residents living in 26 SNFs in an urban Detroit jurisdiction with high SARS-CoV-2 prevalence identified an overall attack rate of 44%, a 37% COVID-19 hospitalization rate, and a 24% fatality rate amid ongoing and widespread SARS-CoV-2 transmission. Repeated point prevalence surveys enabled early identification of COVID-19 cases (including asymptomatic patients), informed cohorting and IPC practices, and guided prioritization of health department resources.

Despite barriers to implementing rapid repeated point prevalence surveys, this assessment demonstrates benefits of conducting repeated surveys in SNFs. Among facilities participating in both surveys, the percentage of new laboratory-confirmed cases declined from 35% to 18%, suggesting that facility-wide testing and on-site IPC support might have contributed to reductions in SARS-CoV-2 transmission. Following testing and establishment of a COVID-19 care unit, IPC assessment and consultation were critical to assisting facilities in targeting interventions to mitigate suspected causes of ongoing transmission. These included incomplete resident and health care personnel cohorting, continued reintroduction of the virus (e.g., from admission of residents with unknown COVID-19 status or residents requiring routine outpatient medical treatment, such as hemodialysis), and space limitations prohibiting use of private rooms to isolate residents whose infection status was unknown. Repeated point prevalence surveys might also improve patient outcomes by enabling earlier identification and initiation of clinical patient monitoring (e.g., assessing vital signs more frequently) and, when warranted, rapid transfer to acute care facilities.

The findings in this report are subject to at least four limitations. First, although asymptomatic health care personnel with SARS-CoV-2 infection are a likely source of transmission, health care personnel were not tested on the same day as were residents, and results of health care personnel testing were not available for inclusion in this report. Second, the long testing interval might influence interpretation of results. The first point prevalence survey occurred approximately 1 month after SARS-CoV-2 introduction in most facilities; therefore, asymptomatic cases identified during the first survey might represent residents who recovered from illness but still had positive RT-PCR test results. Further, the 14-day interval between the two surveys might have resulted in less effective case identification than a shorter interval would have. Third, testing methods in the two surveys varied, as did test characteristics across different platforms and specimen sources (*6*). Finally, at the time of manuscript drafting, data for repeated point prevalence surveys were available for only 12 out of 26 facilities, which limited our ability to fully describe ongoing SARS-CoV-2 transmission among Detroit SNFs.

When repeated point prevalence surveys are implemented as part of COVID-19 response strategies in SNFs, testing results should inform prompt and specific actions, such as 1) using transmission-based precautions for resident care and excluding health care personnel with positive test results from work; 2) strict cohorting of residents and health care personnel; 3) active clinical monitoring of confirmed COVID-19 cases; 4) managing safe transitions of care to and from outside facilities; and 5) discontinuing transmission-based precautions if a test-based strategy is used (*7*,*8*). In response to a confirmed case, CDC now recommends repeat testing (e.g., every 3–7 days) of all residents and health care personnel who previously had negative test results until testing identifies no new cases of COVID-19 among residents or health care personnel (*9*). Widescale testing activities should be integrated with intensified IPC support from local and state health departments.

Repeated point prevalence surveys coupled with IPC support might have reduced SARS-CoV-2 transmission in SNFs in Detroit and have the potential to improve outcomes among SNF residents. New cases continued to be identified during the second survey; however, reductions in 21-day hospitalization and mortality rates were observed throughout the implementation period. Future studies of COVID-19 in SNFs should further explore the impact of repeated point prevalence surveys on morbidity and mortality, the role of asymptomatic health care personnel in SARS-CoV-2 transmission, and the role of serologic testing in reopening SNFs following outbreaks. As the availability of SARS-CoV-2 testing increases, repeated point prevalence surveys and intensified IPC support from public health practitioners are essential components of COVID-19 IPC strategies in SNFs experiencing COVID-19 outbreaks.

**Summary**

**What is already known about this topic?**

Symptom-based screening in skilled nursing facilities (SNFs) is inadequate to detect SARS-CoV-2 transmission. Repeated point prevalence surveys can identify asymptomatic cases during outbreaks.

**What is added by this report?**

Repeated point prevalence surveys at 26 Detroit SNFs identified an attack rate of 44%; within 21 days of diagnosis, 37% of infected patients were hospitalized and 24% died. Among 12 facilities participating in a second survey and receiving on-site infection prevention and control (IPC) support, the percentage of newly identified cases decreased from 35% to 18%.

**What are the implications for public health practice?**

Repeated point prevalence surveys in SNFs can identify asymptomatic COVID-19 cases, inform cohorting and IPC practices, and guide prioritization of health department resources.

## Footnotes

*
https://www.ecfr.gov/cgi-bin/retrieveECFR?gp=&SID=83cd09e1c0f4c6937cd9d7413160fc3f&pitd=20180719&n=pt44.1.46&r=PART&ty=HTML
